# An In Vitro Anti-Cancer Activity of *Ocimum tenuiflorum* Essential Oil by Inducing Apoptosis in Human Gastric Cancer Cell Line

**DOI:** 10.3390/medicina57080784

**Published:** 2021-07-30

**Authors:** Wongwarut Boonyanugomol, Kamolchanok Rukseree, Pornpan Prapatpong, Onrapak Reamtong, Seung-Chul Baik, Myunghwan Jung, Min-Kyoung Shin, Hyung-Lyun Kang, Woo-Kon Lee

**Affiliations:** 1 Department of Sciences and Liberal Arts, Amnatcharoen Campus, Mahidol University, Amnatcharoen 37000, Thailand; Kamolchanok.ruk@mahidol.edu; 2 Department of Public Health, Amnatcharoen Campus, Mahidol University, Amnatcharoen 37000, Thailand; Pornpan.pra@mahidol.edu; 3 Department of Molecular Tropical Medicine and Genetics, Faculty of Tropical Medicine, Mahidol University, Bangkok 10400, Thailand; Onrapak.rea@mahidol.edu; 4 Department of Microbiology, Gyeongsang Institute of Health Sciences, College of Medicine, Gyeongsang National University, Jinju 52727, Korea; scbaik@gnu.ac.kr (S.-C.B.); mjung@gnu.ac.kr (M.J.); mkshin@gnu.ac.kr (M.-K.S.); kangssi@gnu.ac.kr (H.-L.K.); wklee@gnu.ac.kr (W.-K.L.)

**Keywords:** *Ocimum tenuiflorum*, essential oil, gastric cancer, cell migration, cell invasion, apoptosis

## Abstract

*Background and Objectives*: The effects of *Ocimum tenuiflorum* essential oil (OTEO) against gastric cancer remain unknown and merit investigation. *Materials and Methods*: In the present study, the anti-cancer activity of OTEO was examined in a human gastric cancer cell line (AGS). After OTEO treatment, AGS cell viability was determined by an MTT assay, and inhibition of metastasis was determined by cell migration and invasion assays. The expression of apoptosis-related genes in treated AGS cells was determined by qRT-PCR. *Results*: OTEO significantly decreased AGS cell viability in a dose-dependent manner (IC_50_ 163.42 µg/mL) and effectively inhibited cell migration and invasion. Morphological examination demonstrated that OTEO induced cell shrinkage, chromatin condensation, and fragmentation, which are considered typical morphologies of apoptotic cell death. Pro-apoptotic genes (*TP53*, *BAX*, and *BAK*) were significantly up-regulated, while anti-apoptotic genes (*BCL-2* and *BCL-xL*) were significantly down-regulated after treatment with OTEO. In addition, significantly increased gene expression was detected for *CASP8*, *CASP9*, and *CASP3* in AGS cells exposed to OTEO. GC-MS analysis demonstrated that the major compound of OTEO was caryophyllene (25.85%) and α-pinene (11.66%). *Conclusions*: This in vitro study demonstrates for the first time that OTEO has potential anti-gastric cancer activity and may induce apoptosis in AGS cells through extrinsic and intrinsic pathways.

## 1. Introduction

Gastric cancer is one of the most common cancers worldwide, and was the fourth leading cause of cancer-related mortalities in 2020 [1]. The incidence of gastric cancer has wide geographical variations, but it is the major cancer in East Asian countries such as Japan, China, and Korea [2,3]. Although gastric carcinogenesis has been associated with several risk factors, *Helicobacter pylori* (*H. pylori*) infection is well-known as being the major contributor to risk, particularly in East Asian populations. The gold standard for gastric cancer treatment remains surgical resection, but survival rates vary between early-stage and advanced-stage patients [4]. In the treatment of advanced-stage patients, chemotherapeutic drugs are beneficial for relieving symptoms and reducing the rate of metastasis [5]. Currently, the most effective regimens for advanced-stage treatment of this cancer involve epirubicin, cisplatin, or 5-fluorouracil [6]. However, undesirable adverse effects have been documented in gastric cancer patients treated with cisplatin or 5-fluorouracil including nausea, vomiting, and hematologic toxicity. These may affect patients’ quality of life [7]. Therefore, the investigation of agents derived from medicinal plants that exhibit effective anti-cancer activity which are inexpensive and are safe or have minimal side effects represents an interesting option that may lead to further improvement of gastric cancer treatment regimens.

Notably, more than 80% of developed drug compounds come from medicinal plant origins. Throughout the world, many such plants have been used as traditional medicines since ancient times. These plants contain many phytochemical agents that may be extracted in some measure from any part including leaves, flowers, barks, roots, and fruits. *Ocimum tenuiflorum* L. (synonym *Ocimum sanctum*), or holy basil, belonging to the family Lamiaceae, is widely distributed in Thailand and several other countries. *O. tenuiflorum* leaves are popularly used by local populations as functional foods or vegetable foods and are widely used by traditional practitioners for the treatment of various diseases such as bronchitis, malaria, diarrhea, and skin disorders [8]. During the last two decades, pre-clinical studies in animal models have revealed potential multi-pharmacological effects of *O. sanctum* crude extracts including anti-diabetic, wound healing, anti-oxidant, and anti-microbial activity [8]. Additionally, there is accumulating in vitro evidence that the ethanolic extract of *O. sanctum* has significant anti-proliferative activity and could induce apoptosis in cancer cells, including against lung and prostate cancer cell lines [9,10].

In recent years, attention has focused on essential oils. These extracts capture the secondary metabolites of plants, consisting primarily of terpenes but also other diverse chemical components. Several essential oils have been documented to possess anti-cancer effects by means of mechanisms such as anti-mutagenic activity, detoxification enhancement, anti-oxidant activity, and induction of apoptosis [11]. Although the effects of *O. tenuiflorum* essential oil (OTEO) have been studied in some cancer cell lines, and it has been shown to trigger apoptosis mechanisms [12], there is no data specifically concerning the anti-cancer and apoptosis induction activities of OTEO on human gastric cancer cells. Hence, in the present study, we firstly determined the effect of OTEO with respect to its anti-cancer activity against a human gastric cancer cell line (AGS) and then elucidated the associated mechanisms. The foundational data obtained from this study nominate this medicinal plant for further development as a chemotherapeutic agent, especially against gastric cancer.

## 2. Materials and Methods

### 2.1. Extraction of O. tenuiflorum Essential Oil (OTEO)

Holy basil was purchased from a vegetable market in the Amnatcharoen province in northeastern Thailand. The essential oil was extracted from fresh leaves of *O. tenuiflorum* using the hydro-distillation method with a Clevenger apparatus. Excess water was removed with anhydrous sodium sulfate. The pale-yellow essential oil was kept in a small brown bottle, tightly sealed and stored at −20 °C until use. The OTEO was solubilized by absolute ethanol for stock solution preparation (100 mg/mL). Botanical identification of this plant was performed and a voucher specimen was deposited at the herbarium of the Pharmaceutical Botany Department, Mahidol University (PBM 005560).

### 2.2. Cell Culture

The human gastric cancer cell line AGS (ATCC CRL-1739) was provided by the Department of Microbiology, Gyeongsang Institute of Health Science, Gyeongsang National University College of Medicine, Republic of Korea. This cell line was maintained in RPMI medium 1640 (Gibco, Life Technologies Corp., Grand Island, NY, USA) supplemented with 10% fetal bovine serum (FBS) (Gibco, Life Technologies Ltd., Paisley, UK), 100 µg/mL of streptomycin, and 100 U/mL of penicillin (Gibco, Life Technologies Corp., Grand Island, NY, USA). The cells were cultured at 37 °C in a humidified 5% CO_2_ atmosphere.

### 2.3. Determination of Cell Viability

AGS cells were cultured in 96-well plates at a density of 1.5 × 10^4^ cells/well. After 24 h of incubation, the culture media was removed and the cells were washed three times with 1X phosphate buffered saline (PBS). Then, the cells were treated with OTEO at a range of concentrations prepared using 2-fold serial dilution and incubated at 37 °C (5% CO_2_) for 24 h. Afterward, the medium was discarded and 50 µL of 5 mg/mL Thiazolyl Blue Tetrazolium Bromide (MTT) solution (Sigma, St. Louis, MO, USA) was added before further incubation for 4 h (at 37 °C in a 5% CO_2_ incubator). Finally, the MTT solution was removed and precipitated formazan crystals were solubilized with dimethyl sulfoxide (DMSO) (100 µL/well). The quantity of solubilized formazan crystal was determined from the absorbance at 570 nm as measured by a microplate reader (SPECTROstar NANO, BMG Labtech, Germany). The percentage of viable cells was compared with vehicle-treated control cells (0.5% ethanol). The inhibitory concentration 50% (IC_50_) of OTEO was calculated using the AAT Bioquest IC_50_ calculator (AAT Bioquest, Inc., Sunnyvale, CA, USA). All determinations were performed in triplicate as independent experiments.

### 2.4. Cell Migration Assay

AGS cells were cultured in 12-well plates at a density of 1.5 × 10^5^ cells/well and incubated at 37 °C under a humidified 5% CO_2_ atmosphere for 24 h. Then, the confluent monolayer of cells was wounded using a 20–200 µL pipette tip and subsequently washed with 1X PBS to remove unattached cells. Following wound formation, cells were treated with OTEO at the IC_50_ concentration for 24 h. Control cells were cultured with RPMI 1640 without OTEO. Finally, images were captured under an inverted microscope (ECLIPSE Ts2-FL, Nikon, Tokyo, Japan) and the width of the cell-wound-free area was analyzed by NIS Elements Imaging Software version 4.60 (Nikon, Tokyo, Japan). Percent cell migration was calculated by comparing the remaining cell-free area with the area of the initial wound. This assay was performed in triplicate as independent experiments.

### 2.5. Cell Invasion Assay

Cell invasion assays were performed using the QCM ECMatrix Cell Invasion Assay kit (ECM550) (EMD Millipore Corp., Billerica, MA, USA) according to the manufacturer’s instructions. Briefly, RPMI 1640 containing 20% FBS was added to the lower chamber of a 24-well plate. An invasion chamber containing an 8-µm polycarbonate membrane layered with ECMatrix^TM^ was aseptically inserted into the 24-well plate. AGS cells were prepared in serum-free media containing OTEO (IC_50_), then 300 µL of the cell suspension (1 × 10^6^ cells/mL) was subsequently added into the invasion chamber. Serum-free medium without OTEO was used as control. The 24-well plate was incubated at 37 °C for 24 h in a CO_2_ incubator, after which non-invading cells were removed from the invasion chamber and the invasive cells were stained with crystal violet for 20 min. Stained invasive cells at the lower surface of the invasion chamber were observed and counted under an inverted microscope (ECLIPSE Ts2-FL, Nikon, Tokyo, Japan).

### 2.6. Determination of Cell Death and Nuclear Morphology

Briefly, AGS cells cultured in a six-well plate (3 × 10^5^ cells/well) were treated with OTEO at the IC_50_ concentration for 24 h. Then, an inverted microscope (ECLIPSE Ts2-FL, Nikon, Tokyo, Japan) was used to observe cell death morphology in the treated cells and in untreated control cells.

In order to determine nuclear morphology, AGS cells adhered on a cell culture slide were treated with OTEO (IC_50_) for 24 h, or were pre-treated for 2 h with the pan-caspase inhibitor z-VAD-fmk (50 µM) (Sigma, St. Louis, MO, USA) before treatment with OTEO (IC_50_) for 24 h. The treated cells were subsequently stained with 2.5 µg/mL of 4′,6-diamidino-2-phenylindole dihydrochloride (DAPI) (Sigma, St. Louis, MO, USA), their nuclear morphology was observed under a fluorescence microscope (BX53F2, Olympus, Tokyo, Japan), and the observations compared with untreated control cells. Apoptotic cells exhibiting chromatin condensation or fragmentation were enumerated out of a total of 500 evaluated cells.

Briefly, AGS cells grown on a cell culture slide were treated with OTEO (IC_50_) for 24 h. DNA fragmentation of these cells was determined using the TUNEL Assay Kit (ab206386) according to manufacturer’s instructions (Abcam, Cambridge, UK) and observation of cells under a light microscope (CX33RTFS2, Olympus, Tokyo, Japan). Apoptotic cells expressing DNA fragmentation presented a dark brown signal, while lighter shades of brown and/or blue-green to green-brown indicated non-reactive or negative cells.

### 2.7. Apoptosis-Related Gene Expression by Quantitative Reverse Transcription Polymerase Chain Reaction (qRT-PCR)

AGS cells were seeded into a six-well plate at a density of 3 × 10^5^ cells/well and grown at 37 °C under a humidified 5% CO_2_ atmosphere for 24 h. Then, the culture medium was removed and the cells were washed three times with 1X PBS before treatment with OTEO (IC_50_) and incubation for 1, 3, 6, and 12 h. Cells cultured in medium without OTEO were used as the control group. At each indicated timepoint, total RNA was extracted using the Ribozol^TM^ RNA Extraction Reagent (Amresco, VWR, Solon, OH, USA) according to the manufacturer’s instructions. The concentration and purity of RNA extracted from the samples were quantified using a UV-vis spectrophotometer (NanoDrop^TM^ One^c^, Thermo Fisher Scientific, Waltham, MA, USA). cDNA synthesis was performed using the cDNA synthesis kit (Vivantis, Malaysia) according to the manufacturer’s instructions. Expression was determined for the apoptosis-related genes *BCL-2*, *BCL-xL*, *TP53*, *BAX*, *BAK*, *CASP8* (Caspase-8), *CASP9* (Caspase-9), and *CASP3* (Caspase-3); primer sequences are listed in Table 1 and were reported in previous studies [13,14,15,16,17,18,19]. Briefly, each PCR reaction consisted of SYBR Green Realtime PCR master mix (TOYOBO, Osaka, Japan), 0.5 µM primers, and 1 µL of cDNA. PCRs were performed in a LightCycler^®^ 96 real-time PCR instrument (Roche Diagnostics GmbH, Germany) with the following program: 95 °C for 10 min, then 40 cycles of 95 °C for 30 s, 55 or 60 °C for 30 s, and 72 °C for 30 s. Cycle threshold (Ct) values for each gene were normalized to *GAPDH*. Relative expression of apoptosis-related genes was quantified using the 2^−∆∆Ct^ method in comparison to untreated control cells.

### 2.8. Gas Chromatography-Mass Spectrometry Analysis (GC-MS)

The chemical compositions of OTEO were analyzed by using a gas chromatography-mass spectrometry (GC-MS) equipped with a PerkinElmer Headspace Therbo matrix 40 auto-sampler (Clarus 690, Perkin Elmer, Waltham, MA, USA). A capillary column of 30 m × 0.25 mm i.d. Elite-5MS column with 0.25 µm film thickness (Perkin Elmer, Waltham, MA, USA) was used for gas chromatographic separation. The OTEO sample contained in a 22 mL headspace vial was heated at an equilibrium temperature of 90 °C for 10 min, and the gas phase were injected into GC-MS for analysis. The injection time was 0.10 min with constant mode.

GC conditions were as follows. The injector was maintained at 280 °C in a split mode (10:1). The column oven temperature was initially set at 60 °C for 1 min and then increased by 4 °C/min until 280 °C where it was held at this temperature for 5 min. Helium was used as the carrier gas and set a constant flow rate of 1 mL/min. MS detection was carried out at 200 °C and required in the electron impact (EI) mode, using the full scan mode from *m*/*z* 30 to 600 with a scanning speed at low level. The identification of volatile compounds was based on a comparison of their GC retention time and mass spectra with the reference mass spectra from the US National Institute of Standards and Technology (NIST 2017) with more than 75% similarity.

### 2.9. Statistical Analysis

The data are presented as mean ± SD from independent triplicate experiments. Student’s *t*-test was used to determine the significance of differences between treated and control groups. A *p*-value less than 0.05 was considered statistically significant.

## 3. Results

### 3.1. OTEO Inhibits AGS Cell Viability

**Figure 1:**
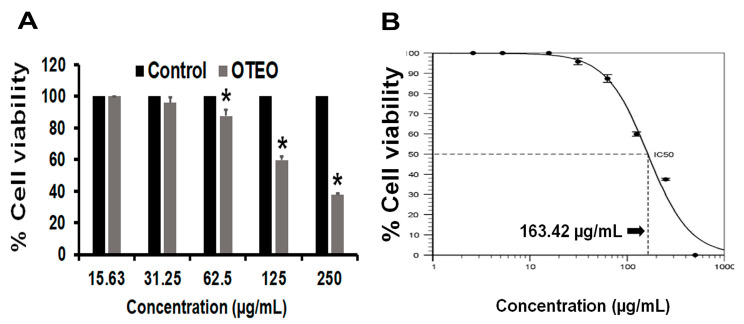
(**A**) Percentage of viable AGS cells after treatment with OTEO for 24 h, as measured by MTT assay. (**B**) Dose-response curve and IC_50_ value of OTEO in the AGS cell line determined using AAT Bioquest IC_50_ calculator. Data are represented as mean ± SD from triplicate independent experiments. * *p*-values less than 0.05 considered significant when compared with control.

The effect of OTEO on AGS cell viability was determined by an MTT assay (Figure 1). At 24 h after treatment with OTEO at 62.50, 125, and 250 µg/mL, 87.4%, 59.83%, and 37.47% of AGS cells were viable relative to the control cells (*p* < 0.05). We found that 500 µg/mL of OTEO completely inhibited AGS cell viability. All told, OTEO inhibited AGS cell viability in a dose-dependent manner, with an IC_50_ value of 163.42 µg/mL as calculated by the AAT Bioquest IC_50_ calculator.

### 3.2. OTEO Inhibits Migration and Invasion of AGS Cells

**Figure 2:**
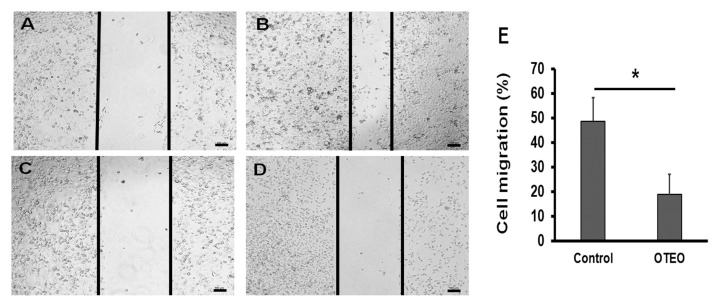
Representative images from wound-healing assays indicating the inhibition of cell migration induced by OTEO in AGS cells: (**A**) AGS control at 0 h, (**B**) AGS control at 24 h, (**C**) AGS treated with OTEO at 0 h, and (**D**) AGS treated with OTEO at 24 h. (**E**) Percent cell migration was calculated by comparing the width of the remaining cell-free area with the width of the initial wound. Data are shown as mean ± SD. Scale bars indicate 100 µm. * *p* < 0.05 indicated significant difference relative to the control group.

**Figure 3:**
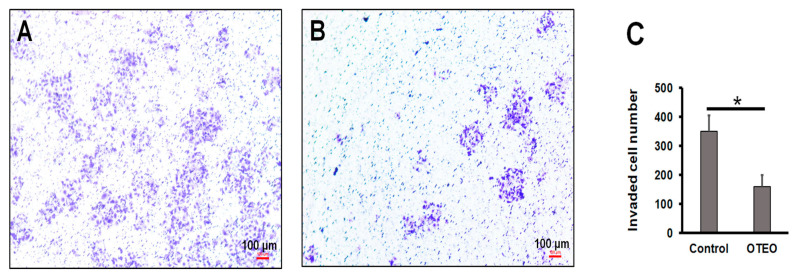
OTEO inhibits invasion by AGS cells, determined using the QCM ECMatrix Cell Invasion Assay kit: (**A**) AGS control cells, (**B**) AGS cells treated with OTEO, and (**C**) enumeration of invasive cells. Data are shown as mean ± SD. Scale bars indicate 100 µm. * *p* < 0.05 indicated significant difference relative to the control group.

Cell migration was evaluated by in vitro wound healing assay, which is a standard assay for determining anti-migration activity by agents in cancer cell lines. As shown in Figure 2, cell migration was substantially reduced in AGS cells treated with OTEO (18.6% at 24 h), when compared to untreated control cells (48.7% at 24 h). Similarly, using an in vitro assay of cell invasion, we found that the number of invasive AGS cells was significantly decreased after treatment with OTEO at 24 h when compared with the control (*p* < 0.05) (Figure 3).

### 3.3. Characterization of Cell Death and Nuclear Morphology in AGS Cells

**Figure 4:**
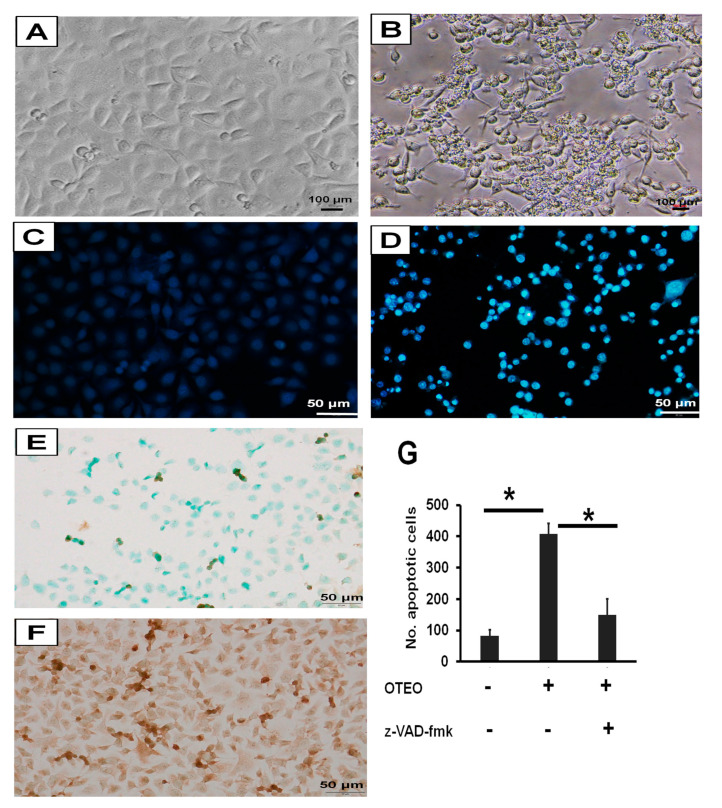
Determination of cell and nuclear morphology in AGS cells. (**A**,**B**) Cell morphology observed under inverted microscope. (**C**,**D**) Nuclear morphology determined by DAPI staining. (**E**,**F**) DNA fragmentation examined by TUNEL assay. (**A**,**C**,**E**) AGS untreated control, (**B**,**D**,**F**) AGS treated with OTEO (IC_50_). (**G**) Enumeration of apoptotic cells with DAPI staining. Data are shown as mean ± SD. Scale bars indicate 100 µm (**A**,**B**) or 50 µm (**C**–**F**). * *p* < 0.05 indicates significant difference when compared with control group or z-VAD-fmk treatment.

To investigate the mechanism associated with cell death in AGS cells treated with OTEO (IC_50_), they were first observed cell and nuclear morphology at 24 h after treatment. Cell shrinkage, membrane blebbing, and apoptotic bodies were readily evident in AGS-treated cells (Figure 4A,B), which constitute the typical morphology of apoptotic cell death. Chromatin condensation in apoptotic AGS cells was observed using DAPI staining assays at 24 h after treatment with OTEO (Figure 4C,D). A significant increase of apoptotic cells was detected in cultures exposed to OTEO when compared with the control group (Figure 4G). In contrast, pre-treatment of AGS cells with z-VAD-fmk resulted in a significant decrease of apoptotic cells relative to OTEO treatment alone (Figure 4G). To confirm apoptosis-induced DNA fragmentation, TUNEL-positive cells were quantified, with high numbers resulting for AGS cells treated with OTEO (Figure 4E,F). These data indicate that OTEO may exert anti-cancer activity against AGS cells through the mechanism of apoptosis.

### 3.4. Effects of OTEO on Apoptosis-Related Gene Expression in AGS Cells

**Figure 5:**
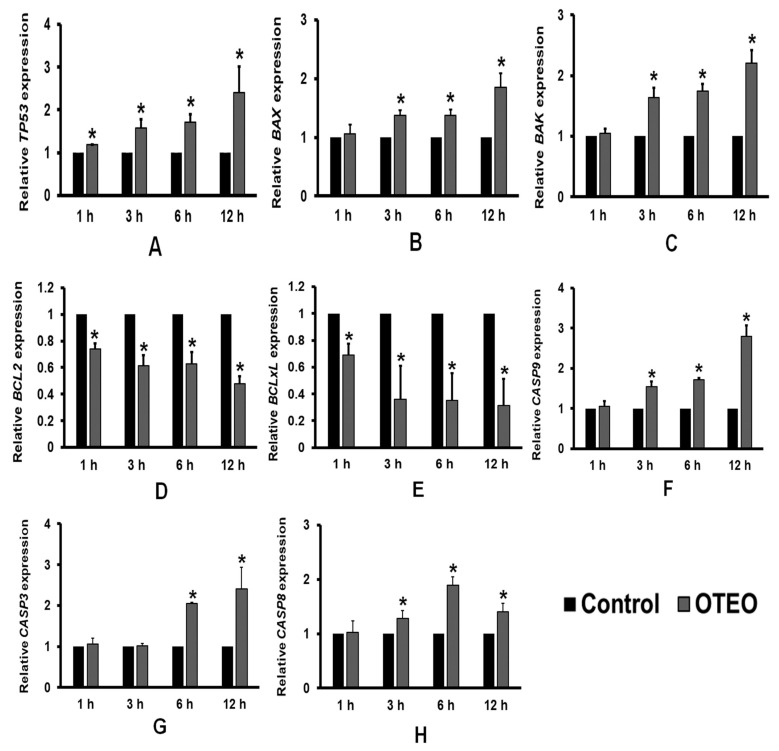
Effect of OTEO on the gene expression of (**A**) *TP53*, (**B**) *BAX*, (**C**) *BAK*, (**D**) *BCL-2*, (**E**) *BCL-xL*, (**F**) *CASP9*, (**G**) *CASP3*, and (**H**) *CASP8*, in AGS treated cells, quantified by qRT-PCR. All data are presented as mean ± SD of the gene expression relative to an untreated control group, which were examined in triplicate independent experiments. * *p* < 0.05 indicates statistical significance versus control.

To further investigate the anti-cancer mechanism of OTEO associated with apoptosis, qRT-PCR was used to determine the expression of apoptosis-related genes (Figure 5). AGS cells were treated with OTEO (IC_50_) for 1, 3, 6, and 12 h, and the expression of selected genes was quantified. After OTEO treatment, expression of the pro-apoptotic genes *TP53*, *BAX*, and *BAK* was significantly increased (Figure 5A–C). Conversely, expression of anti-apoptotic genes (*BCL-2* and *BCL-xL*) was found to be significantly decreased in AGS cells treated with OTEO (Figure 5D,E). Finally, OTEO treatment significantly stimulated expression of the genes encoding Caspase-9 (*CASP9*), Caspase-8 (*CASP8*), and Caspase-3 (*CASP3*) (Figure 5F–H).

### 3.5. Chemical Composition of OTEO

The volatile composition from OTEO was determined by GC-MS analysis and the results are shown in Table 2. A total of 25 compounds belonging to monoterpenes (37.11%), sesquiterpenes (32.18%), oxygenated monoterpenes (13.53%), and others (17.18%) were identified. GC-MS analysis revealed that the major compound of OTEO was caryophyllene (25.85%) followed by α-pinene (11.66%). Several minor compounds (1–10% area) of OTEO were also identified, such as camphene (9.37%), eucalyptol (8.26%), β-pinene (5.96%), eugenol (4.70%), β-elemene (4.68%), methyl eugenol (4.25%), estragole (3.28%), D-limonene (3.11%), linalyl acetate (3.01%), isoborneol (2.03%), α-terpineol (2.03%), β-ocimene (1.86%), O-cymene (1.60%), β-phellandrene (1.54%), and camphor (1.21%).

## 4. Discussion

Several chemotherapeutic agents derived from medicinal plants have been investigated for potential development of cancer treatments. *O. tenuiflorum* is a medicinal plant widely distributed in South and Southeast Asia that is commonly used as a vegetable food. Interestingly, the essential oil extracted from this plant exerts biological properties beneficial for human health including anti-microbial, anti-inflammatory, and anti-cancer activities. Its specific potential as an anti-gastric cancer agent has not been investigated. Accordingly, in the present study, we investigated the anti-cancer activity and induction of apoptosis by OTEO in a human gastric cancer cell culture (AGS) model.

OTEO exerted strong cytotoxicity in AGS cells in a dose-dependent manner, with the IC_50_ value being 163.42 µg/mL. The IC_50_ dose was used in subsequent experiments of this study. Notably, cancer cell migration and invasion are important for cancer metastasis, which is the mechanism of cancer cell spread to distant organs [20]. Gastric cancer has been found to disseminate to organs such as the liver, colon, or lungs [21,22,23], and it has been proposed that distant organ metastasis indicates a poor prognosis [24]. Our in vitro findings demonstrate that OTEO is able to suppress the migration and invasion of AGS cells. However, the underlying molecular signaling by which OTEO suppresses AGS cell metastasis needs to be further elucidated. Several previous studies have shown cytotoxic and anti-metastasis activity of *O. tenuiflorum* extracts on a variety of cancer cell lines. The ethanolic extract of *O. sanctum* (EEOS) demonstrated growth inhibitory activity against several cancer cell types including human non-small cell lung carcinoma (A549) [10,25], prostate cancer cells [9], and head and neck squamous cell carcinoma (HNSCC) [26]. Similarly, Manaharan et al. found that the purified essential oil from *O. sanctum* (OSEO) inhibited cell proliferation of human breast cancer cells (MCF-7); their IC_50_ value of 170 µg/mL was similar to our result [12]. In addition, Kwak et al. demonstrated through molecular analysis that the anti-metastatic mechanism of EEOS is mediated by the inhibition of phosphatidylinositide 3-kinases (PI3K)/Akt in non-small cell lung cancer cells [27]. Meanwhile, treatment of HNSCC with EEOS resulted in a significant inhibition of cell invasion due to attenuating the activity of matrix metalloproteinase (MMP)-2 and MMP-9 [26]. Our in vitro results clearly extend the significant inhibitory effect of OTEO treatment on cancer cell viability and metastasis to human gastric cancer cells.

Apoptosis is a programmed cell death response elicited by several stimuli and is an essential process of tissue homeostasis [28]. This pathway contributes to the elimination of unnecessary or expired cells of normal tissues. However, cancer cells usually evade this pathway, leading to uncontrolled cell proliferation [29]. It is well documented that the tumor suppressor protein p53 plays an important role in inducing apoptosis when cells are exposed to stress or harmful conditions [30]. Under stress conditions, p53 is stimulated and associates with apoptosis signals enacted through both intrinsic and extrinsic pathways [31]. The intrinsic pathway of apoptosis is initiated by mitochondrial membrane permeabilization, which is regulated by members of the Bcl-2 protein family including anti-apoptotic proteins such as Bcl-2, Bcl-x_L_, and Mcl-1and pro-apoptotic proteins such as Bax, Bad, Bak, and Bid [32]. The inhibition of anti-apoptotic proteins leads to Bax/Bak oligomerization on the mitochondrial membrane, causing a release of apoptogenic proteins into the cytosol (these include cytochrome c and SMAC/Diablo). The released cytochrome c specifically binds to the cytosolic protein APAF-1, facilitating assembly of the apoptosome that subsequently induces Caspase-9 and Caspase-3 activation, leading to apoptotic cell death [33]. Meanwhile, the extrinsic pathway is associated with ligand-mediated trimerization of death receptors in the tumor necrosis factor family, which subsequently activates Caspase-8, leading to a cleavage of downstream effector caspases (Caspase-9 or -3) [34]. In this study, AGS cells treated with OTEO exhibited irregular morphology such as cell shrinkage, membrane blebbing, chromatin condensation, and DNA fragmentation, which constitute the typical morphology of apoptotic cell death. Additionally, pre-treatment of cells with the pan-caspase inhibitor z-VAD-fmk rescued this morphology, demonstrating that OTEO may induce apoptosis in AGS cells through caspase activity.

In further characterization of the role of OTEO in apoptosis, qRT-PCR assays revealed that OTEO treatment of AGS cells potentially decreased gene expression levels of *BCL-2* and *BCL-xL*, while significantly elevating *TP53*, *BAX*, and *BAK*. Likewise, increased gene expression of *CASP9* (Caspase-9) and *CASP3* (Caspase-3) was detected in AGS cells treated with OTEO, the protein products of which are eventually linked to the execution phase of apoptosis. These data suggested that OTEO may be associated with mitochondrial membrane dysfunction and subsequent induction of apoptosis in AGS cells. In addition, our study has also shown that gene expression of components of the *CASP8* (Caspase-8)-mediated extrinsic pathway was significantly increased after treatment with OTEO. Several lines of evidence likewise indicate effects of *O. tenuiflorum* extracts on the induction of apoptosis in other cancer cells. Our findings are consistent with a previous study showing that EEOS effectively induced apoptosis in human prostate cancer cells (LNCaP) through the down-regulation of BCL-2 and significant elevation of Caspase-9 and Caspase-3 activities in a dose-dependent manner [9]. Another previous study similarly reported that OSEO increased the proportion of apoptotic cells by up-regulating *TP53*, *BID*, and the BAX/BCL-2 ratio in human breast cancer cells (MCF-7) [12]. In the present study, we indicate that OTEO may induce apoptotic cell death in gastric cancer cells through both intrinsic and extrinsic pathways.

An essential oil can contain 20–60 chemical constituents with varying concentrations, but only two or three are primary constituents [11]. The chemical constituents of OTEO might additionally vary depending on geography or environment conditions. Our preliminary GC-MS study identified caryophyllene and α-pinene as the major chemical constituent of OTEO, followed by camphene, eucalyptol, β-pinene, eugenol, β-elemene, methyl eugenol, etc. This list had partial similarity to a previous report studying the aromatic profiles of essential oils from *Thai basils* [35]. Notably, each of the major and minor constituents in OTEO may be isolated and tested independently as a pure compound for anti-cancer activity against human gastric cancer cells, which may help in finding the most effective active ingredient for use in gastric cancer treatment. However, it has been suggested that the blend of molecules in an essential oil is more potent than its isolated constituents due to synergism or antagonism, resulting in more selective effects [36]. Accordingly, this should also be considered during the further development of essential oil products.

## 5. Conclusions

In conclusion, this is the first in vitro study to demonstrate that OTEO treatment significantly inhibits cell viability and metastasis in gastric cancer cells (AGS). OTEO treatment of AGS cells led to cell death, which may be mediated by *TP53* activation inducing apoptosis through both intrinsic and extrinsic pathways. This in vitro work provides fundamental knowledge regarding the mechanism of action of OTEO anti-gastric cancer activity. However, the effects of OTEO on other signaling pathways in gastric cancer cells require further study, and determination of its synergism with chemotherapeutic drugs is also recommended.

## Figures and Tables

**Table 1:** PCR primer sequences used in qRT-PCR.

Gene	Primers	Annealing (°C)
*GAPDH*	Forward: 5′-TCATCAGCAATGCCTCCTGCA-3′	55
	Reverse: 5′-TGGGTGGCAGTGATGGCA-3′	
*BCL* *-* *2*	Forward: 5′-CAGGATAACGGAGGCTGGGATG-3′	60
	Reverse: 5′-AGAAATCAAACAGAGGCCGCA-3′	
*BCL* *-* *xL*	Forward: 5′-ACCCCAGGGACAGCATATCA-3′	60
	Reverse: 5′-TGCGATCCGACTCACCAATA-3′	
*TP53*	Forward: 5′-TAACAGTTCCTGCATGGGCGGC-3′	55
	Reverse: 5′-AGGACAGGCACAAACACGCACC-3′	
*BAX*	Forward: 5′-TGGCAGCTGACATGTTTTCTGAC-3′	60
	Reverse: 5′-TCACCCAACCACCCTGGTCTT-3′	
*BAK*	Forward: 5′-ATGGTCACCTTACCTCTGCAA-3′	60
	Reverse: 5′-TCATAGCGTCGGTTGATGTCG-3′	
*CASP8*	Forward: 5′-AGAGTCTGTGCCCAAATCAAC-3′	60
	Reverse: 5′-GCTGCTTCTCTCTTTGCTGAA-3′	
*CASP9*	Forward: 5′-CGAACTAACAGGCAAGCAGC-3′	60
	Reverse: 5′-ACCTCACCAAATCCTCCAGAAC-3′	
*CASP3*	Forward: 5′-GCGGTTGTAGAAGAGTTTCGTG-3′	60
	Reverse: 5′-CTCACGGCCTGGGATTTCAA-3′	

qRT-PCR:Quantitative Reverse Transcription Polymerase Chain Reaction.

**Table 2:** Chemical composition of the essential oil extracted from *O. tenuiflorum*.

Retention Time (min)	Compounds	%Area
4.61	2,5-Diethyltetrahydrofuran	0.95
5.22	3-Carene	0.53
5.42	α-Pinene	11.66
5.81	Camphene	9.37
6.31	β-Phellandrene	1.54
6.46	β-Pinene	5.96
6.66	β-Myrcene	0.69
7.65	O-Cymene	1.60
7.79	D-Limonene	3.11
7.90	Eucalyptol	8.26
8.23	β-Ocimene	1.86
8.63	γ-Terpinene	0.39
9.82	Linalyl acetate	3.01
11.35	Camphor	1.21
11.90	Isobornyl thiocyanoacetate	0.99
12.15	Isoborneol	2.03
12.48	γ-Terpinene	0.40
12.90	α-Terpineol	2.03
12.97	Estragole	3.28
17.95	Eugenol	4.70
18.76	Copaene	0.88
19.17	β-Elemene	4.68
19.44	Methyl eugenol	4.25
20.18	Caryophyllene	25.85
21.22	Humulene	0.77

## Data Availability

Data sharing is not applicable.
